# The Anti-tuberculosis Battle in Greece in the 1800s and 1900s

**DOI:** 10.7759/cureus.26023

**Published:** 2022-06-16

**Authors:** Spyros N Michaleas, Athanase D Protogerou, Nikolaos V Sipsas, George Panayiotakopoulos, Angelos-Charidimos Angelakis, Christina Michailidou, Marianna Karamanou

**Affiliations:** 1 Department of History of Medicine and Medical Ethics, National and Kapodistrian University of Athens School of Medicine, Athens, GRC; 2 Department of Pathophysiology, National and Kapodistrian University of Athens School of Medicine, Athens, GRC; 3 Department of General Pharmacology, Patras University, Patras, GRC

**Keywords:** phrenicotomy, sanatoria, artificial pneumothorax, bcg vaccine, robert koch

## Abstract

Tuberculosis is an infectious disease that mainly affects the lungs (known as pulmonary tuberculosis). *Mycobacterium tuberculosis* is a species of pathogenic bacteria in the family of Mycobacteriaceae and the causative agent of tuberculosis; it was discovered by Robert Koch in 1882. From about 1918 to 1939, tuberculosis in Greece was characterized as a social disease because it seemed to spread among the lower social classes, including displaced people living in refugee camps. The battle against tuberculosis involved private initiatives aimed at educating people on hygiene and establishing anti-tuberculosis institutions, such as sanatoria and preventoria.

## Introduction and background

The term *tuberculosis *was introduced in 1834, although Hippocrates (460 BC-377 BC) was the first to describe the disease. The root word “tubercle” comes from the Latin *tuberculum*, a diminutive of the word “tuber,” meaning swelling or lump. The term describes the pus-filled abscesses that form in the lungs of a person infected by pulmonary tuberculosis, the most common form of the disease [1-3]. Until the mid-1800s, the prevailing theory was that tuberculosis was hereditary. This misperception likely resulted from observing its rapid and highly contagious spread among households with children. From 1880 to 1890, scientists began to understand more about the disease, including how it is transmitted [4]. The main purpose of this article is to highlight the anti-tuberculosis battle in Greece from the mid-19th century to the 20th century.

## Review

Tuberculosis: transmission and diagnosis

**Figure 1:**
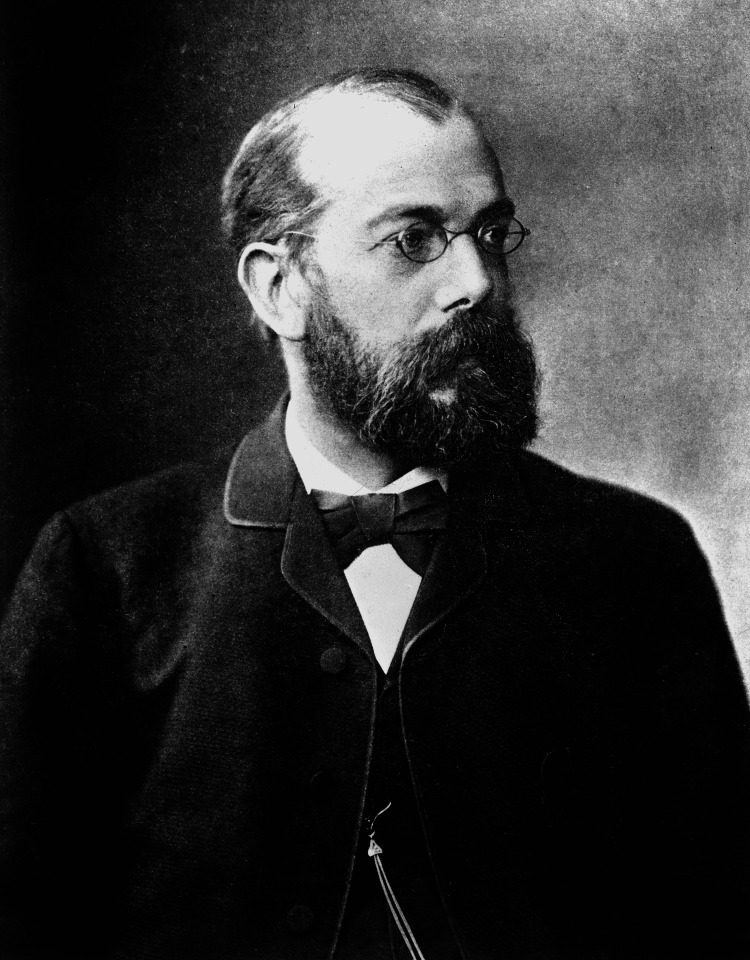
Robert Koch Permission has been granted to use the image. Credit: Portrait of Robert Herman Koch (1843-1910), bacteriologist. Wellcome Collection. Attribution 4.0 International (CC BY 4.0).

Tuberculosis is an infectious disease transmitted to humans via *Mycobacterium tuberculosis*, or “Koch bacillus,” named after the German researcher Robert Heinrich Hermann Koch (1843-1910) (Figure 1), who identified its structure and method of cultivation in a lecture in Berlin on March 24, 1882. He received the Nobel Prize in Physiology or Medicine in 1905 for this discovery [5-8]. The disease can be acquired directly or indirectly [1]. Direct contact involves inhalation of infected droplets from a sick person, such as through coughing, sneezing, laughing, loud talking, or close contact. Indirect contact involves airborne transmission via contaminated sputum [9], fluids, secretions, feces, or animal secretions [6].

A common belief in the 19th century was that tuberculosis could be suspended in dust and dirt [9]. Fabrics and clothing, such as carpets, curtains, and women’s dresses, even the air outside, were considered vehicles of disease [10,11]. As a result, households were discouraged from sweeping as a cleaning method and instead recommended wiping surfaces with a damp cloth [10]. Until the middle of the 20th century, many believed that tuberculosis could be transmitted through the digestive tract by consuming meat, milk, or dairy products from animals infected by the bovine tuberculosis bacterium (*Mycobacterium bovis*) [6,7,12,13].

Tuberculosis can affect any organ of the human body, although it mainly affects the lungs. The most common symptoms are fever, intense sweating, anorexia, fatigue, and weight loss, which are ambiguous symptoms that can make diagnoses difficult. Tuberculosis also is typically associated with the formation of granulomas and, in the case of pulmonary tuberculosis, intense coughing, expectoration, shortness of breath, and hemoptysis [13-15].

**Figure 2:**
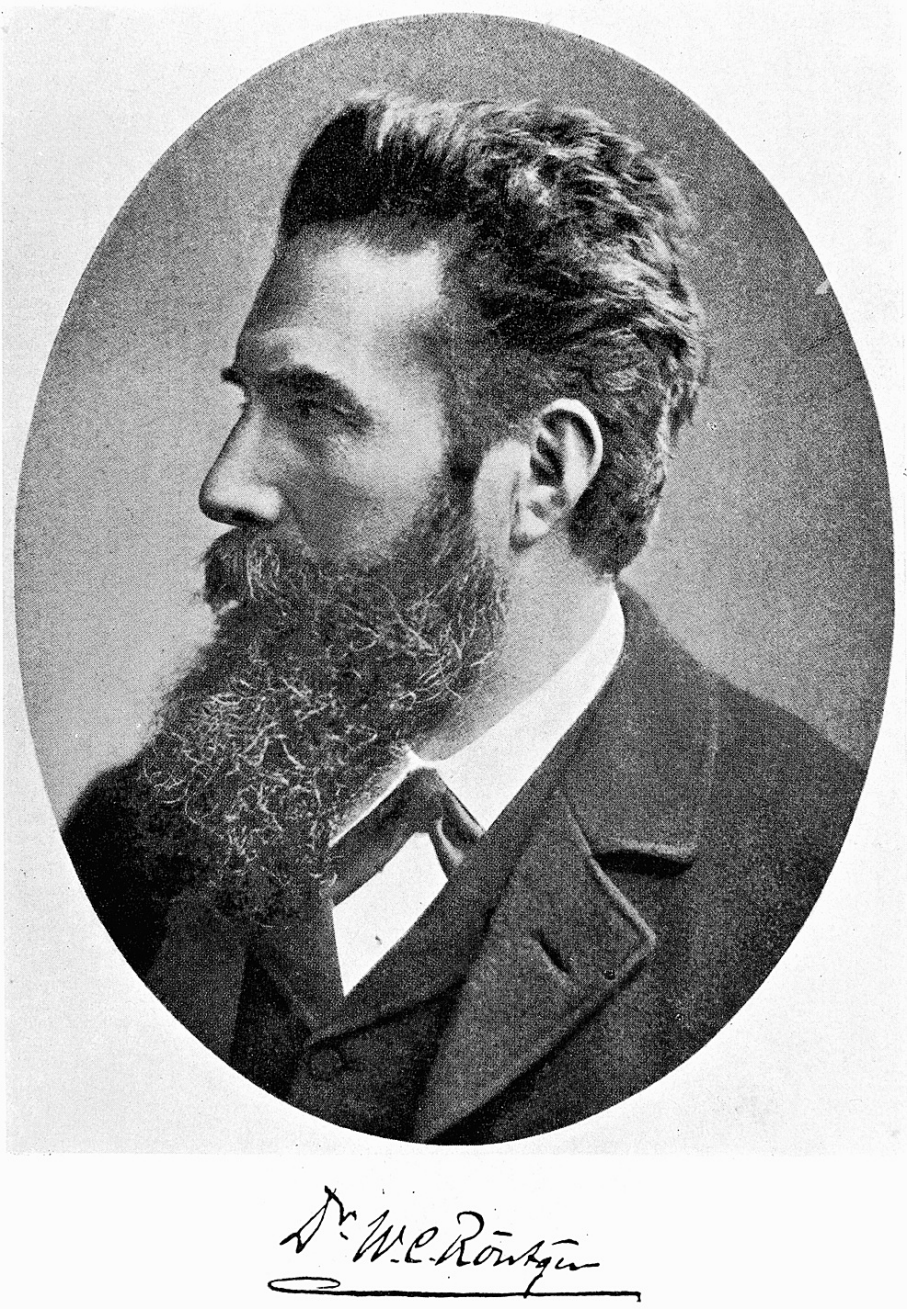
Wilhelm Conrad Röntgen Permission has been granted to use the image. Credit: Portrait of W. C. Roentgen. Wellcome Collection. Attribution 4.0 International (CC BY 4.0).

The discovery of X-rays in 1895 by the German physicist Wilhelm Conrad Röntgen (1845-1923) (Figure 2) and the invention of the bronchoscope in 1898 by German laryngologist Gustav Killian (1860-1921) contributed greatly to clinical diagnostic methods [2,13,14]. Dr. Charles Mantoux (1877-1947) also devised a basic method for diagnosing tuberculosis using tuberculin. The Mantoux method was a popular tool because it did not require special laboratory equipment. Nowadays, microbiological polymerase chain reaction (PCR) tests are used to confirm suspected cases of many diseases, including tuberculosis [13].

Treatment

**Figure 3:**
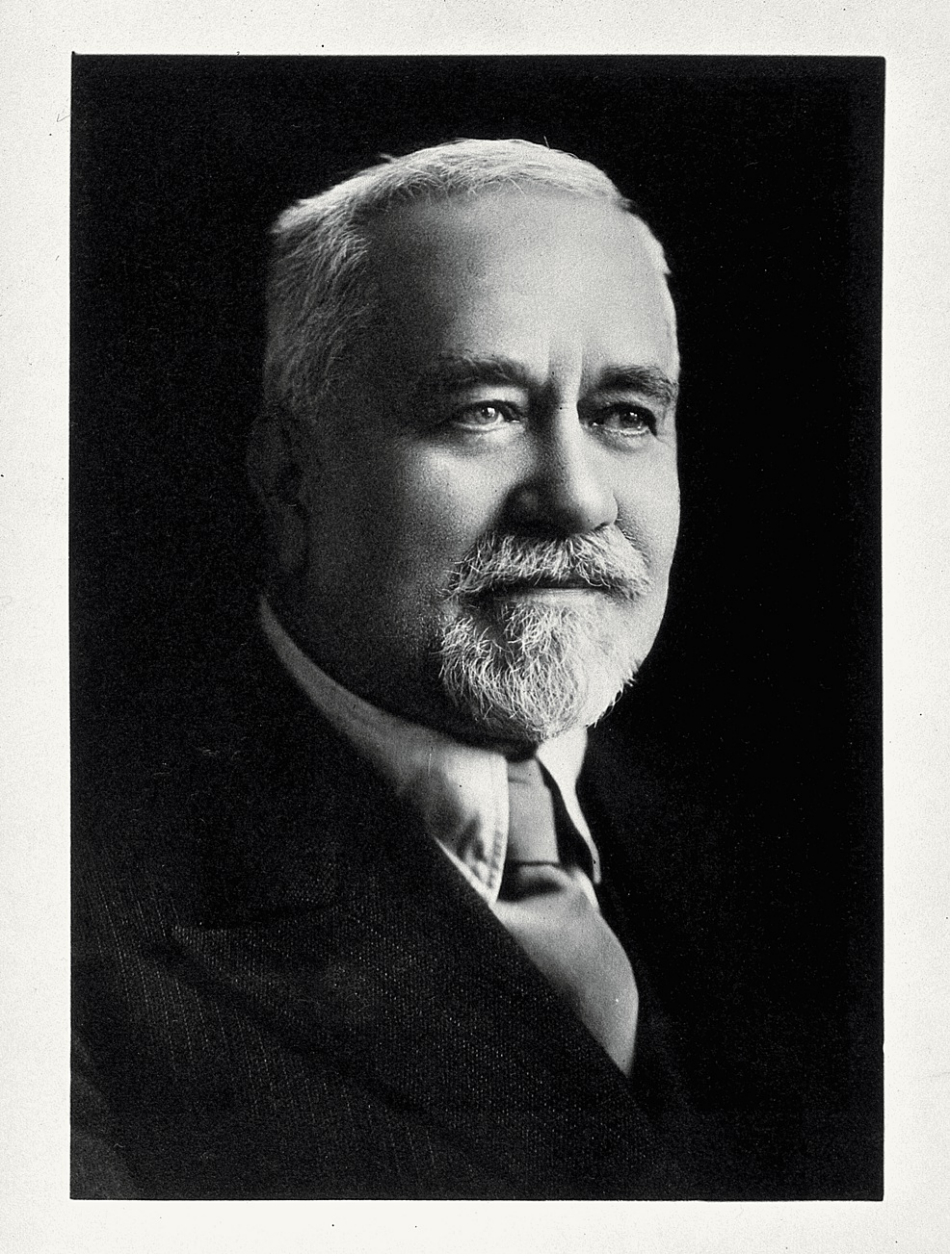
Léon Charles Albert Calmette Permission has been granted to use the image. Credit: Albert Calmette. Photograph. Wellcome Collection. Public Domain Mark.

In the early 1900s, the Pasteur Institute in Lille, France, conducted several experiments on the antigenic potency of mycobacterial strains and found that they had no virulence [7]. As a result, in 1919, the French tuberculosis researcher Léon Charles Albert Calmette (1863-1933) (Figure 3) collaborated with veterinarian Camille Guérin (1872-1961) to culture the Calmette-Guérin bacillus, which eventually was developed into the Bacille de Calmette et Guérin (BCG) vaccine [2,7,8,13]. The BCG vaccine was first administered in France in 1921 as an oral agent and then intradermally. In 1924, the Pasteur Institute provided the vaccine free of charge and freely shared instructions on how to produce it with microbiology laboratories [2,8].

Over the years, various other therapies were developed. The main perception among scientists was that early diagnosis of the disease was essential for effectively treating the disease [6,16,17]. By the middle of the 20th century, scientific studies had shifted focus to education, including disseminating information on the prevention of tuberculosis and other noninvasive treatments [4,18]. For example, the hygienic and dietetic method involved long-term treatment in sanatoria or hospital asylums focusing on strengthening the body’s defenses and shielding it from anything that could spread disease to other organs. This method promoted physical rest, peace of mind, and fresh air in combination with a nutritious diet [2,3,7-10,14,19-21].

**Figure 4:**
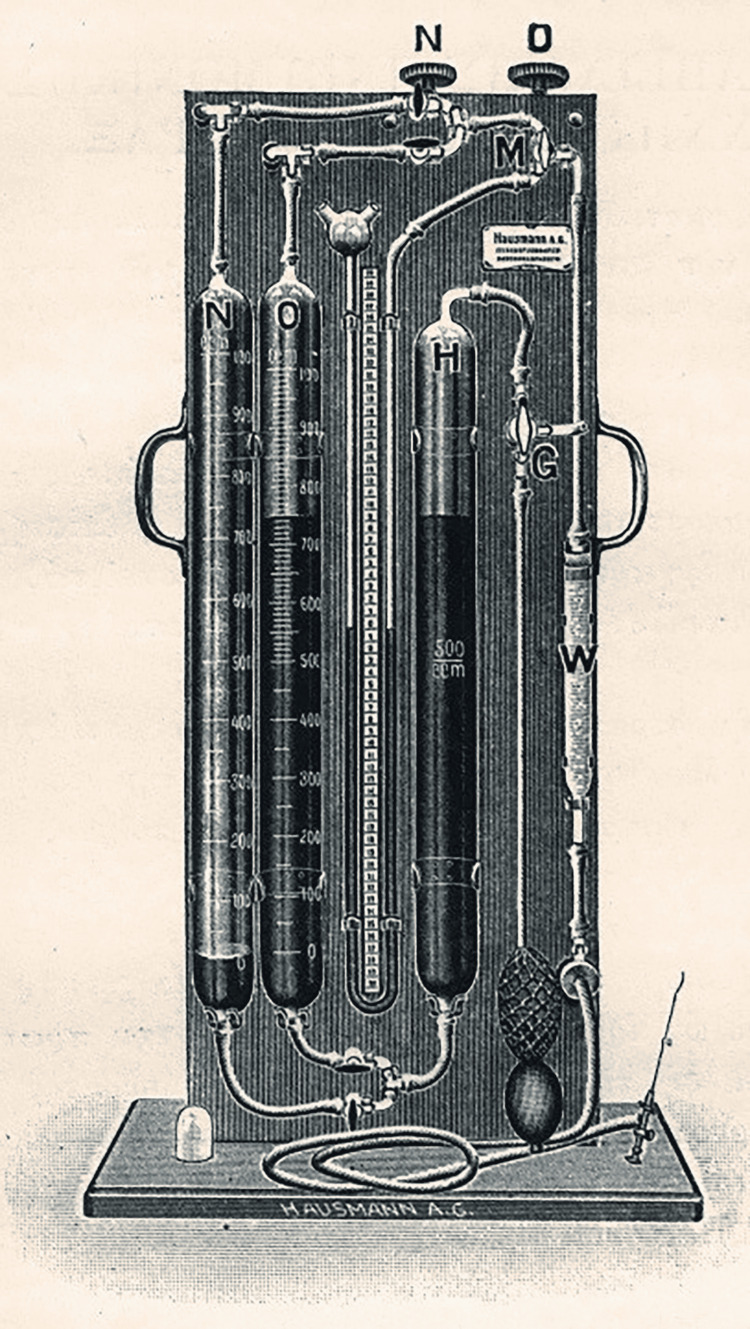
Artificial pneumothorax device Permission has been granted to use the image. Source: Panagiotakos P: Tuberculosis and sanatoria (early diagnosis and treatment of pulmonary tuberculosis) (Book in Greek). BG Teubner, Leipzig; 1922 [3].

More invasive procedures were available for serious cases. Italian physician Carlos Forlanini (1847-1918) initiated the widespread use of the artificial pneumothorax in 1892 [3,5] (Figure 4). This syringe device injected oxygen, nitrogen gas, or atmospheric air into the pleural cavity via a mercury cylinder [2,3,7-9]. From 1920 to 1940, the device was used to help reduce lung damage caused by tuberculosis, and the procedure was repeated every three to four weeks as the patient underwent continuous X-rays to check the progression of the disease [2,4,16,17,19]. Another procedure, the phrenicotomy (phrenic resection/frenectomy), was proposed in 1911 by Austrian physician Ernst Stuertz (1870-1942). It was performed in cases of pleural effusion with the aim of paralyzing and raising the diaphragm [8]. Another invasive method, thoracoplasty, resulted in a permanent alteration of the shape of the chest wall [2].

Tuberculosis in Greece

Since the establishment of the Greek state in 1830, the effort to treat tuberculosis is based primarily on measures to reduce the transmission of the disease. In particular, with the Royal Decree of 1836 “on the prevention of the transmission of infectious diseases” (Government Gazette 28/31.12.1836), tuberculosis was recognized as a contagious disease.

The fight against tuberculosis in Europe, including Greece, was based on three basic parameters: detection, isolation, and prophylaxis. An indirect goal was to educate the population on prevention based on the medical knowledge of the time. During the Interwar Period in Greece, tuberculosis was considered a social disease. When the illness was detected, the infected were banished, but the disease continued to affect all age groups, particularly young people aged 10 to 30 years. Refugee camps and other crowded locations suffered multiple outbreaks.

The rapid spread of the disease in the 19th century and unsuccessful efforts at developing a cure led the medical community to take mainly protective measures. The medical knowledge of that time indicated a correlation between tuberculosis transmission and unsanitary living conditions, such as in the neighborhoods of the lower social classes. However, the disease should affect even the higher social classes who probably chose to be admitted to a private sanatorium in Greece or abroad [1,3,8,12,16,19,20,22]. Lack of adequate nutrition also was considered a factor in disease spread [5,7,16]. Anti-tuberculosis campaigns thus focused their efforts on reforming workplaces so that fresh air could be circulated in cramped, sunless, and humid areas [13]. In tobacco factories, combustible gases were found to be too dangerous for workers’ health [8].

Vasilios Patrikios (1847-1929) was a physician who had studied in Athens and Paris. At the Hellenic Medical Society’s First Panhellenic Medical Conference in Athens, held in May 1901, he suggested constructing anti-tuberculosis clinics in urban centers, as well as establishing branches in existing hospitals dedicated to the treatment of tuberculosis patients. Patrikios wanted to alert people to public health issues, particularly the importance of disinfection of areas in which tuberculosis patients lived. He also proposed the creation of disinfection centers and suggested the organization of the First Hellenic Conference Against Tuberculosis [2,4,6,8,10,17].

**Figure 5:**
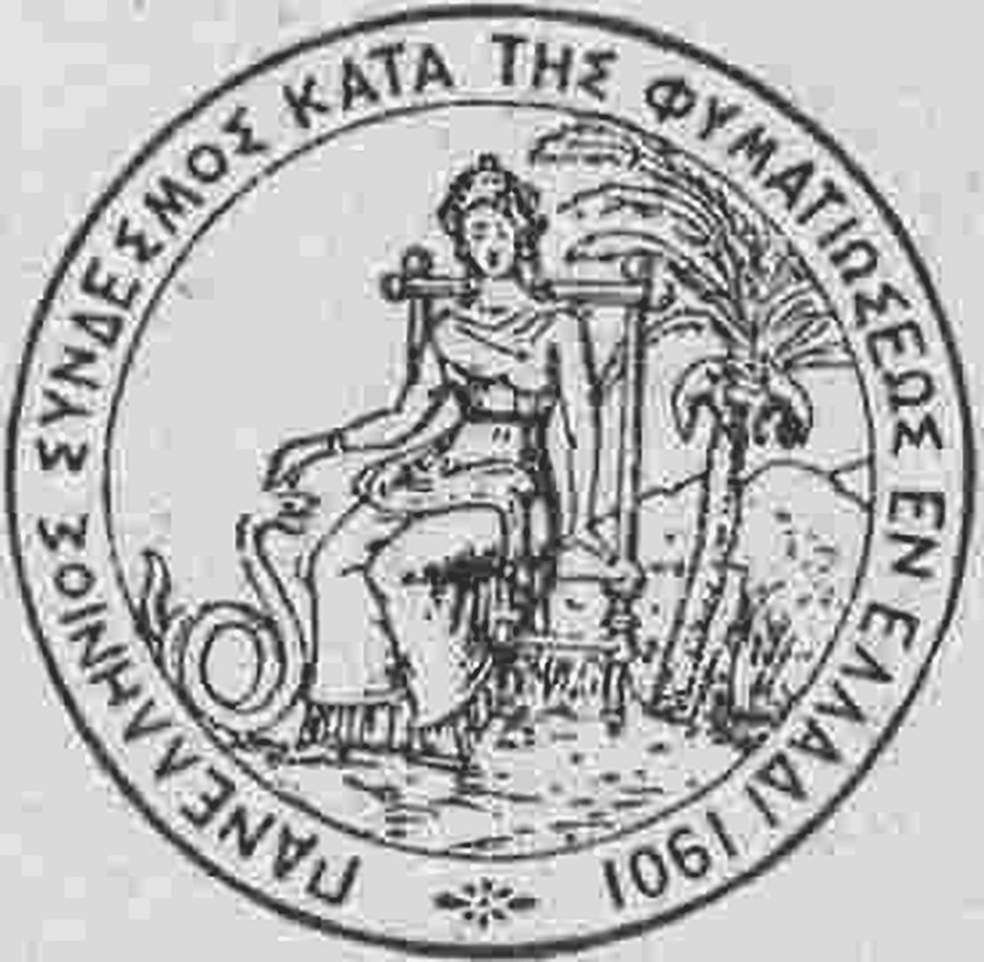
The emblem of the Panhellenic Association Against Tuberculosis Permission has been granted to use the image. Source: Patrikios V: Instructions for protection against tuberculosis (Book in Greek). Estia, Athens; 1902 [6].

As a result of the conference, the Panhellenic Association Against Tuberculosis was established on June 25, 1901 (Figure 5) [23]. The association served two main purposes: eliminate the disease and establish Asclepieions (sanatoria) in Greece [5,8]. It also served as a public health educator, providing information about the current medical research, suggestions for improving living and working conditions, and tips for reducing transmissions, such as eliminating public spitting and alcohol addiction. The association also published books on tuberculosis [2,4,10].

**Figure 6:**
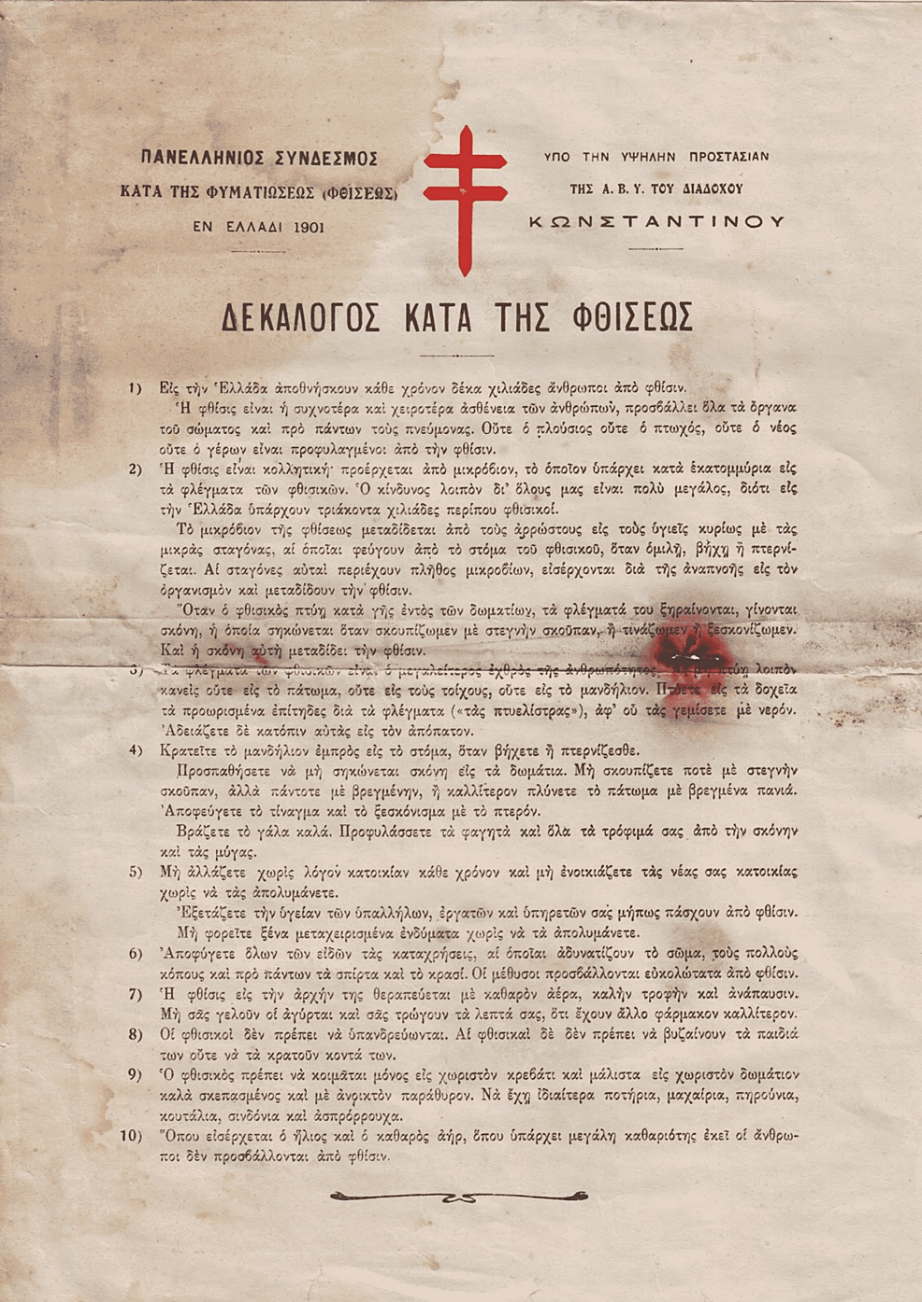
Decalogue Against Tuberculosis Permission has been granted to use the image. Source: Panhellenic Association Against Tuberculosis: The reports of the association (January 1, 1908-December 31, 1909). Athens; 1910 [23].

All the efforts of the association for the protection of the public are also reflected in the “Decalogue Against Tuberculosis” (Figure 6). The leaflet included information about the transmission of the disease, as well as prevention measures. It begins with the reminder that every year, 10,000 people died in Greece from tuberculosis [8,13]. The red cross, which appears in this leaflet, was the symbol of the anti-tuberculosis battle. In fact, it is the “Cross of Lorraine,” which was established as a symbol of the fight against tuberculosis at a conference against tuberculosis in 1902 in Berlin. It refers to the double cross used by Gottfried von Bouillon (1060-1100) during the First Crusade (1096-1099), and for this reason, we can hypothesize that it indicates the determination of the battle [8].

**Figure 7:**
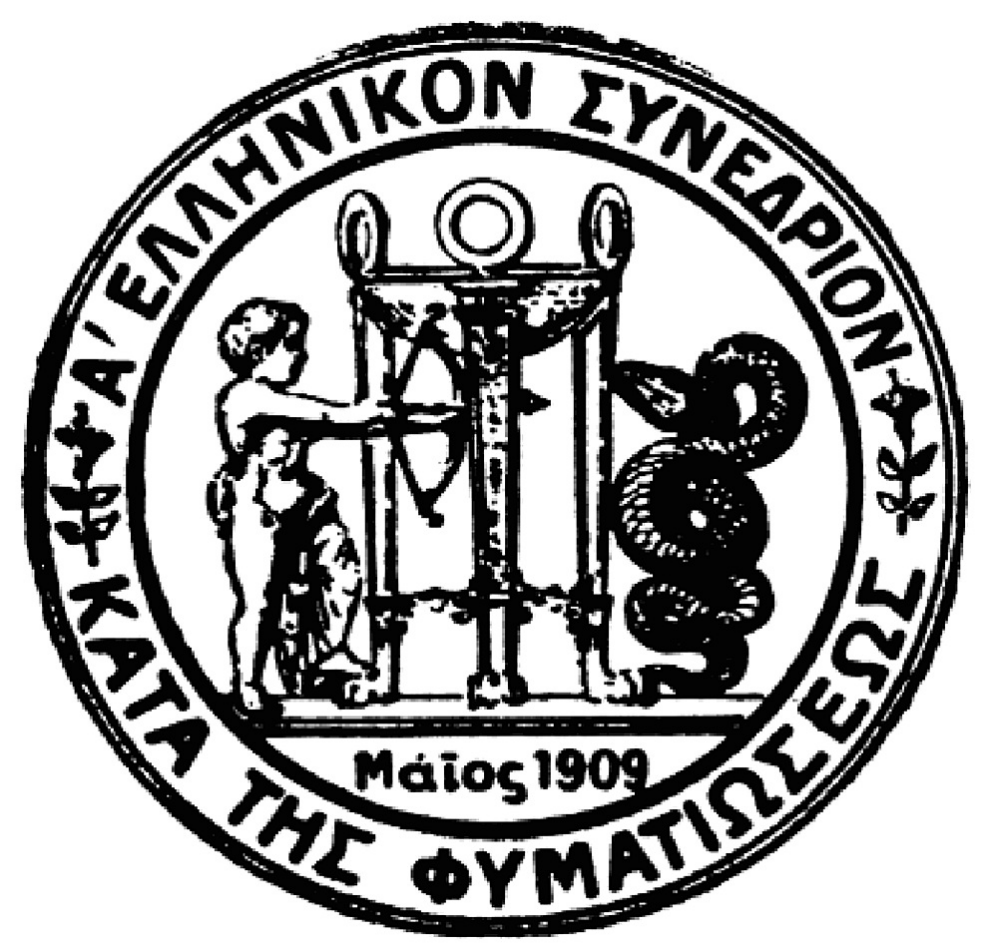
The emblem of the First Hellenic Conference Against Tuberculosis The emblem of the Conference bears Apollo, god of medicine, marking Python with his bow through a tripod. Python symbolizes tuberculosis, while the tripod strengthens the sacred character of the intended purpose, which is none other than the extermination of the “monster.” Permission has been granted to use the image. Source: Kouzis A: Proceedings of the First Hellenic Congress Against Tuberculosis. Chiotis N, Rouseas K, Athens; 1909 [24].

In February 1907, with the support of the Greek Red Cross, the first anti-tuberculosis clinic was founded in Athens. The first artificial pneumothorax procedure was conducted there in 1927 [5]. Moreover, following the proposal of Vasilios Patrikios, the association organized the First Hellenic Conference Against Tuberculosis in 1909 (Figure 7) [24].

Another prominent figure in the fight against tuberculosis was Panagiotis Pampoukis (1858-1956), a pioneering microbiologist who studied with Louis Pasteur in Paris. In 1893, he founded the first microbiological laboratory in Greece, and in 1896, he established an anti-rabies clinic in Athens. Pampoukis was a founding member of the Hygiene Society (1915), president of the Medical-Surgical Society (1924), and founder of the Greek Anti-tuberculosis Society (1925). In 1927, he published a reference book titled *The Battle Against Tuberculosis* to share knowledge about tuberculosis, including measures and methods of treatment for the disease [2,5,7,8,25].

In 1902, the Sotiria Ladies’ Club was founded in Athens, headed by Sophia Schliemann (1852-1932), widow of the archaeologist Henry Schliemann (1822-1890). One of their projects involved working with the Monastery of Petraki to construct a sanatorium in Ymittos in the Goudi area of Athens. In 1905, the first anti-tuberculosis institute, Sotiria, opened [2,14]. It served as an isolation shelter for tuberculosis patients and taught patients about the importance of a healthy diet. These and other preventorias housed children and adolescents suffering from tuberculosis and thus protected their families, neighbors, and other children from contracting the disease [1,12,13]. It should be mentioned that the Sotiria hospital is still in operation and serves as the main hospital for COVID-19 patients.

Moreover, the main legislative work for the organization of the anti-tuberculosis battle during the Interwar Period is the enactment of Law 1979/1920, which was published in the Official Gazette 33/06.2.1920. The provisions of this law refer to the administrative organization of the anti-tuberculosis clinics, hospitals, and sanatoriums [21]. This law seems to have served as a springboard for the construction of sanatoriums and anti-tuberculosis clinics throughout Greece [13].

Greek Prime Minister Ioannis Metaxas (1871-1941) established the Central Committee for the anti-tuberculosis battle around 1936. The main tasks of the committee were to establish 13 anti-tuberculosis institutes and supervise the renovation of the existing sanatoriums. The outbreak of World War II (1939-1945) stalled these efforts, but the discovery of antibiotics and anti-tuberculosis drugs in 1944 soon radically changed these previous treatment methods [5,18].

## Conclusions

In the past, tuberculosis was described as an incurable disease. Until the 1940s, no effective treatment existed. For Greek society in the first decades of the 20th century, tuberculosis was an endemic scourge that decimated much of its population. Its high mortality rate motivated scientists to find therapies and preventive measures. Soon after Robert Koch discovered *Mycobacterium tuberculosis* in 1882, scientists determined that tuberculosis was transmitted via bacterial infection and not hereditary, as previously thought. To stop the spread in Greece, private initiatives worked to educate the public on transmission and prevention and establish anti-tuberculosis institutions for the treatment of those infected with the disease.
